# Identifying HSV-1 Inhibitors from Natural Compounds via Virtual Screening Targeting Surface Glycoprotein D

**DOI:** 10.3390/ph15030361

**Published:** 2022-03-16

**Authors:** Jiadai Wu, Helen Power, Monica Miranda-Saksena, Peter Valtchev, Aaron Schindeler, Anthony L. Cunningham, Fariba Dehghani

**Affiliations:** 1 School of Chemical and Biomolecular Engineering, Faculty of Engineering, The University of Sydney, Sydney 2006, Australia; jiadai.wu@sydney.edu.au (J.W.); helen.power@sydney.edu.au (H.P.); peter.valtchev@sydney.edu.au (P.V.); aaron.schindeler@sydney.edu.au (A.S.); 2 Centre for Advanced Food Engineering, The University of Sydney, Sydney 2006, Australia; 3 Centre for Virus Research, The Westmead Institute for Medical Research, Westmead 2145, Australia; monica.saksena@sydney.edu.au; 4 Bioengineering and Molecular Medicine Laboratory, The Children’s Hospital at Westmead and The Westmead Institute for Medical Research, Westmead 2145, Australia

**Keywords:** herpes simplex virus type 1, virtual screening, molecular docking, glycoprotein D, natural compounds

## Abstract

Herpes simplex virus (HSV) infections are a worldwide health problem in need of new effective treatments. Of particular interest is the identification of antiviral agents that act via different mechanisms compared to current drugs, as these could interact synergistically with first-line antiherpetic agents to accelerate the resolution of HSV-1-associated lesions. For this study, we applied a structure-based molecular docking approach targeting the nectin-1 and herpesvirus entry mediator (HVEM) binding interfaces of the viral glycoprotein D (gD). More than 527,000 natural compounds were virtually screened using Autodock Vina and then filtered for favorable ADMET profiles. Eight top hits were evaluated experimentally in African green monkey kidney cell line (VERO) cells, which yielded two compounds with potential antiherpetic activity. One active compound (1-(1-benzofuran-2-yl)-2-[(5Z)-2H,6H,7H,8H-[1,3] dioxolo[4,5-g]isoquinoline-5-ylidene]ethenone) showed weak but significant antiviral activity. Although less potent than antiherpetic agents, such as acyclovir, it acted at the viral inactivation stage in a dose-dependent manner, suggesting a novel mode of action. These results highlight the feasibility of in silico approaches for identifying new antiviral compounds, which may be further optimized by medicinal chemistry approaches.

## 1. Introduction

Herpes simplex virus type 1 (HSV-1) is a contagious human pathogen that is estimated to affect 3.7 billion people worldwide [1]. Whereas HSV-1 infections are commonly associated with limited facial–oral lesions, severe disease can occur in neonates or immunocompromised individuals (keratitis, meningitis, encephalitis, and disseminated infections) [2,3]. Viral infections are the most common cause of sporadic life-threatening encephalitis in the United States, and up to 75% of these cases are caused by HSV-1 [3,4].

Primary infection with HSV-1 occurs by virus penetration at mucosal surfaces or through skin abrasions. From here, HSV-1 infects innervating sensory nerves of the trigeminal or dorsal root ganglia, where it establishes a life-long latent infection with a high rate of periodical reactivation [2,5,6,7]. The current predominant antiherpetic agents are viral deoxyribonucleic acid (DNA) extension inhibitors, such as acyclovir (ACV), penciclovir, valacyclovir, famciclovir, foscarnet, and cidofovir. However, these drugs only act to shorten the duration of initial and recurrent episodes to a limited degree [8,9,10]. Consequently, HSV-1 infection is usually described as an incurable disease [1]. Drug-resistant HSV-1 strains among immunocompromised patients are a growing health concern and have emerged after decades of exposure to a single class of antiherpetics compounds [11,12,13]. All these issues support the search for new antiviral compounds that target other stages of HSV-1 infection, such as viral attachment and entry. 

Among the 12 surface glycoproteins of HSV, glycoprotein D (gD) plays a key role in the viral attachment and entry process [14,15]. Binding of gD to one of its cellular receptors, nectin-1 or herpesvirus entry mediator (HVEM), induces a conformational change in the structure of gD, which triggers a cascade of molecular interactions, resulting in the formation of a fusion complex involving gH/gL and gB [15,16,17]. Although some steps of the entry process of HSV-1 remain unclear, the critical role of gD in viral binding and entry, as well as its three-dimensional structure in bound and unbound states, has been well-documented [15,17,18,19]. This provides an opportunity to exploit this knowledge using advanced computational approaches to discover novel antiviral compounds that interact with gD.

Naturally derived molecules have been recognized as highly promising sources of novel antiherpetic compounds. The first antiviral drug, Ara-A, was originally derived from the marine compounds: spongonucleosides, spongothymidine, and spongouridine [20]. Subsequently, a broad range of natural compounds have since been found to possess antiviral activities, with half-maximal effective concentration (EC_50_) ranging from 10 to 200 µM. These include emodin (EC_50_ = 21.5 − 195 µM) [21,22,23], epigallocatechin gallate (EC_50_ = 12.5 − 50 µM) [24,25,26,27], curcumin (EC_50_ = 89.6 µM) [28,29,30], and other extracted polyphenol compounds [31]. However, practical screening of bioactive molecules with desirable activity from the large pool of diverse natural compounds can be a costly and time-consuming task. Computational modelling represents an underutilized and efficient alternative to accelerate this complex process. Molecular docking is a well-developed tool for screening promising candidates from libraries of bioactive molecules and has been applied to identify inhibitors of viral targets, including thymidine kinase [29,32,33,34], DNA polymerase [35,36], and protease [37]. However, few molecular docking studies have been attempted with a limited focus on natural compounds and viral surface glycoproteins as the target, suggesting a potential research gap [38,39].

Herein, we present a discovery pipeline to identify novel HSV-1 inhibitors from an extensive natural compound library via virtual screening. Molecular docking was targeted at the HVEM and nectin-1 binding sites of the viral gD to identify compounds that bind the virus and prevent infection with different mechanisms of action than those of current antivirals. The antiviral activity of top-ranked molecules was then investigated using in vitro HSV-1 assays. One natural molecule exhibited antiherpetic activity and could serve as a basis for further drug modification and optimization. These findings highlight the capacity of applying computer-aided techniques to facilitate the discovery of lead active compounds (ACs) with desirable properties.

## 2. Results

### 2.1. Virtual Screening in Search of Potential gD Inhibitors

**Figure 1:**
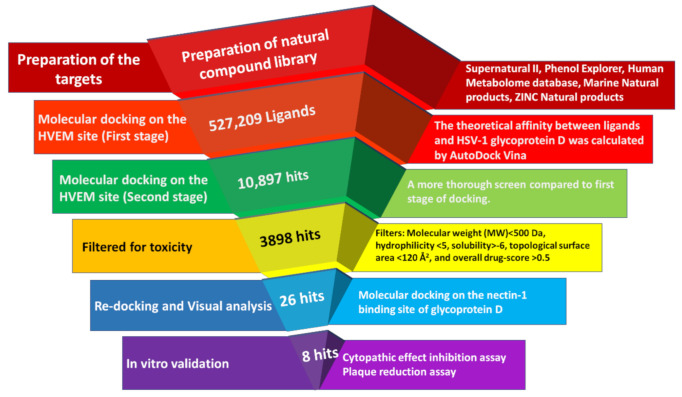
Schematic diagram of the molecular docking workflow for identifying natural glycoprotein D inhibitors with antiviral activity against HSV-1.

The interaction between gD and its two major cellular receptors, nectin-1 and HVEM, can be described as non-reciprocal competitive binding. Glycoprotein D forms a hairpin structure after interacting with HVEM, which masks the binding site for nectin-1 [16,40]. Similarly, the interaction between gD and nectin-1 blocks the HVEM binding sites [16]. As distinct residues interact with each receptor, we performed targeted molecular docking on each site separately. Docking against the HVEM binding site of gD was conducted first, before a refined set of compounds was docked against the nectin-1 binding site. A schematic diagram of the virtual screening steps and outcomes is presented in Figure 1.

AutoDock Vina software was used to estimate the theoretical affinity between the HVEM binding site of gD and a library of 527,209 natural compounds. The generated docking score is based on the change in Gibbs free energy between bound and unbound states; therefore, a more negative score indicates higher putative affinity [41]. A secondary round of screening, which involved performing a greater number of docking runs, was conducted on the top 2% of compounds (*n* = 10,897). These compounds included 6654 molecules from the Supernatural II database, 3813 from ZINC Natural Products, 210 from Human Metabolome, 206 from Marine Natural Products, and 14 molecules from Phenol Explorer. The docking scores from this screen ranged from −10.6 to −4.3. 

To select ACs for downstream in vitro testing, ligands with docking scores ≤−8.2 were filtered for favorable ADMET properties and commercial availability (*n* = 3898). The selection criteria were based on toxicity ratings and drug-conforming behavior according to the OSIRIS property explorer tool [42]. Consequently, 26 drug-like compounds that could be readily obtained from commercial vendors were selected for further molecular docking analysis against the nectin-1 binding site. 

**Figure 2:**
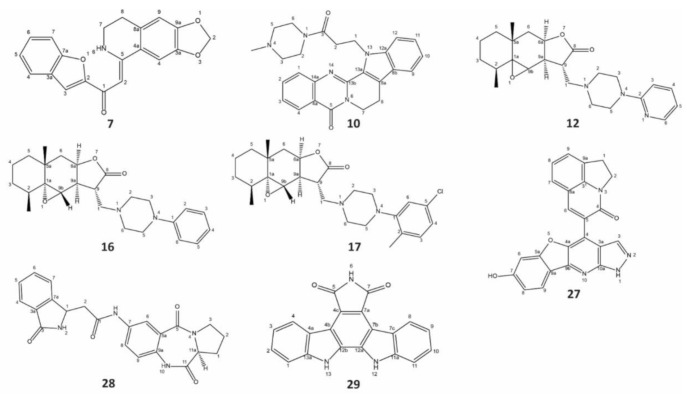
Structure of the eight natural compounds selected as potential glycoprotein D inhibitors by molecular docking. All eight compounds were subjected to in vitro validation. Listed active compounds (ACs) are: #7,1-(1-benzofuran-2-yl)-2-[(5Z)-2H,6H,7H,8H-[1,3]dioxolo[4,5-g]5soquinoline-5-ylidene]ethenone; #10,13-[3-(4-methylpiperazin-1-yl)-3-oxopropyl]-8,13-dihydroindolo[2′,3′:3,4]pyrido[2,1-b]quinazolin-5(7H)-one; #12,(2S,5Ar,6Ar,9S,9Ar)-2,5a-dimethyl-9-((4-(5soquino-2-yl)piperazin-1-yl)methyl)octahydro-2H-oxireno[2′,3′:4,4a]naphtho[2,3-b]furan-8(9Bh)-one; #16,(1Ar,2S,5Ar,6Ar,9S,9Ar,9Bs)-2,5a-dimethyl-9-((4-phenylpiperazin-1-yl)methyl)octahydro-2H-oxireno[2′,3′:4,4a]naphtho[2,3-b]furan-8(9Bh)-one; #17, (1Ar,2S,5Ar,6Ar,9S,9Ar,9Bs)-9-((4-(5-chloro-2-methylphenyl)piperazin-1-yl)methyl)-2,5a-dimethyloctahydro-2H-oxireno[2′,3′:4,4a]naphtho[2,3-b]furan-8(9Bh)-one,#27,5-(7-Hydroxy-1H-benzofuro[3,2-b]pyrazolo[4,3-e]5soquino-4-yl)-1H-pyrrolo[3,2,1-ij]5soquinol-4(2H)-one; #28, N-((S)-5,11-dioxo-2,3,5,10,11,11a-hexahydro-1H-benzo[e]pyrrolo[1,2-a][1,4]diazepin-7-yl)-2-(3-oxoisoindolin-1-yl)acetamide, #29, Arcyriaflavin A. Their molecular structures were illustrated using ChemDraw 18.2 (PerkinElmer).

**Figure 3:**
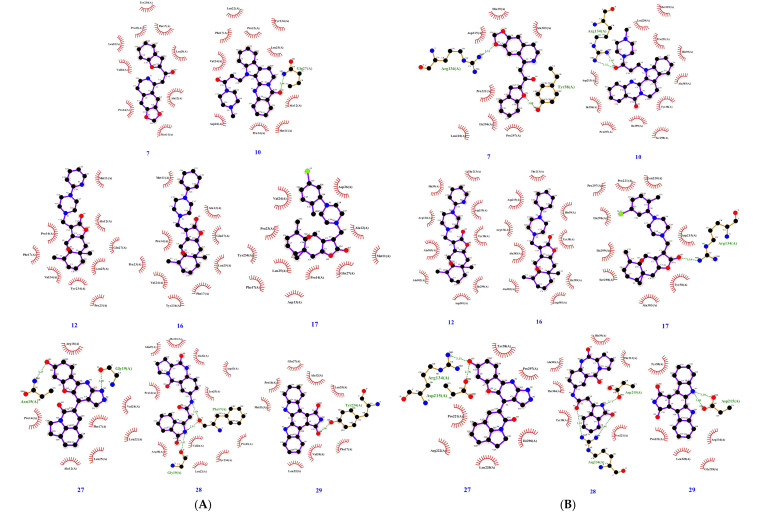
Binding interaction diagrams of the eight compounds selected for in vitro validation with the (herpes virus entry mediator) HVEM (**A**) and nectin-1 (**B**) binding interfaces of HSV-1 glycoprotein D as predicted by molecular docking. Red lines represent hydrophobic contacts, and broken green lines represent hydrogen bonds, with distances in angstroms.

To investigate inhibitory cross-reactivity against the nectin-1 binding site of gD, re-docking analysis was performed with the 26 previously selected compounds. Free energy scores from this redocking screen ranged from −9.4 to −6.8. Docked binding conformations were analyzed by visual inspection, and the top eight ACs with scores ≤−7.6 were selected for in vitro validation. The molecular docking results of the eight selected compounds are listed in Table 1, and their molecular structures are illustrated in Figure 2. The predicted interactions between these eight ligands and the HVEM and nectin-1 binding interfaces of gD are illustrated and outlined in Figure 3 and Table 2. For these eight ligands, the number of predicted interacting residues ranged from 9 to 13 and from 6 to 12 for the HVEM and nectin-1 binding interfaces, respectively. The number of H-bonds ranged from 0 to 3 for both binding interfaces. 

### 2.2. Investigating the Antiviral Activity of Selected Compounds by In Vitro Assays

#### 2.2.1. Determination of Cytotoxicity of ACs

Eight ACs, selected based on their docking scores against both the HVEM and nectin-1 binding interfaces, ADMET profiles, and commercial availability, were investigated for cytotoxicity in African green monkey kidney cell line (VERO). Data from cytotoxicity assays were used to determine test concentrations to be used in cytopathic effect (CPE) inhibition assays, as summarized in Table 3. Three compounds demonstrated negligible toxicity at the highest concentration tested (100 µg/mL) compared with dimethyl sulfoxide (DMSO) control: #7, 1-(1-benzofuran-2-yl)-2-[(5Z)-2H,6H,7H,8H-[1,3]dioxolo[4,5-g]isoquinoline-5-ylidene]ethenone; #27, 5-(7-Hydroxy-1H-benzofuro[3,2-b]pyrazolo[4,3-e]isoquino-4-yl)-1H-pyrrolo[3,2,1-ij]isoquinol-4(2H)-one; and #28, N-((S)-5,11-dioxo-2,3,5,10,11,11a-hexahydro-1H-benzo[e]pyrrolo[1,2-a][1,4]diazepin-7-yl)-2-(3-oxoisoindolin-1-yl)acetamide. These compounds were subsequently tested at 10 µg/mL in downstream CPE assays.

The remaining five ACs exhibited toxicity to the cells at a concentration of 10 µg/mL (Table 3): #10, 13-[3-(4-methylpiperazin-1-yl)-3-oxopropyl]-8,13-dihydroindolo[2′,3′:3,4]pyrido[2,1-b]quinazolin-5(7H)-one; #12, (2S,5Ar,6Ar,9S,9Ar)-2,5a-dimethyl-9-((4-(isoquino-2-yl)piperazin-1-yl)methyl)octahydro-2H-oxiren[2′,3′:4,4a]naphtho[2,3-b]furan-8(9Bh)-one; #16, (1Ar,2S,5Ar,6Ar,9S,9Ar,9Bs)-2,5a-dimethyl-9-((4-phenylpiperazin-1-yl)methyl)octahydro-2H-oxireno[2′,3′:4,4a]naphtho[2,3-b]furan-8(9Bh)-one; #17, (1Ar,2S,5Ar,6Ar,9S,9Ar,9Bs)-9-((4-(5-chloro-2-methylphenyl)piperazin-1-yl)methyl)-2,5a-dimethyloctahydro-2H-oxireno[2′,3′:4,4a]naphtho[2,3-b]furan-8(9Bh)-one; and #29, Arcyriaflavin A. Therefore, these five compounds were tested at a concentration of 1 µg/mL in downstream CPE assays.

#### 2.2.2. Investigating the Mechanism of Action of ACs by Time of Addition Assay

**Figure 4:**
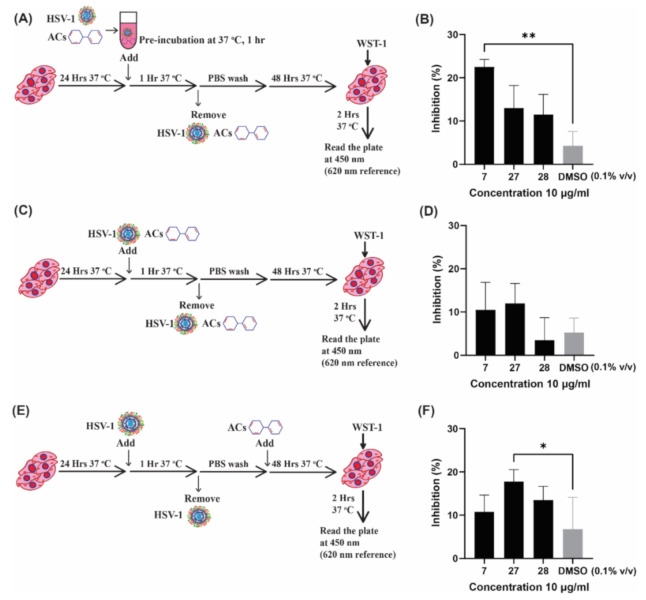
Antiviral screening assays for three selected active compounds. The antiviral activity of active compounds (ACs) was investigated at three different stages. The antiviral activity of ACs #7, #27, and #28 was tested at 10 µg/mL. (**A**) Schematic diagram of the viral inactivation assay performed by preincubating Herpes Simplex Virus type 1 (HSV-1) with ACs for 1 h prior to addition to cells is. (**B**) Results of the viral inactivation assay. Viral attachment and entry inhibition of ACs were investigated by simultaneously adding the virus and ACs to cells, as shown in (**C**); results are shown in (**D**). The post-entry antiviral effect of ACs was tested by adding ACs after infection (**E**); results are plotted in (**F**). Cells infected with the virus and treated with dimethyl sulfoxide (DMSO) at 0.1% *v*/*v* were included as solvent controls. This experiment was performed four times with triplicates. Means of the four experiments are plotted with standard error of the mean. Friedman test, followed by Dunn’s test, was used to determine statistical significance (* *p* < 0.05, ** *p* < 0.01).

Next, the eight ACs were assessed in cell-based assays to test for antiviral activity against HSV-1. Three separate assays were performed to elucidate the mechanism of action (Figure 4A,C,E). A viral inactivation assay was performed by incubating HSV-1 with ACs at 10 µg/mL or 1 µg/mL (depending on cytotoxicity) before addition to the cells. Viral attachment and entry-inhibition assay involved the simultaneous addition of virus and ACs to cells (Figure 4C), whereas post-entry effects of ACs were investigated by adding ACs 1 h post-HSV-1 infection (Figure 4E). The initial screening was performed using a low MOI of 0.01 (Supplementary Materials Figure S1). Three promising ACs, #7, #27, and #28, were selected based on cytotoxicity (Table 3) and preliminary results (Figure S1) and further tested at MOI of 5, which triggered a cytopathic effect in 90% of cultured cells. The inhibitory effect of each compound was measured by cell survival as determined by using water-soluble tetrazolium 1 (WST-1) reagent (Figure 4).

Two out of eight tested compounds demonstrated promising antiviral activity at different stages of infection. AC#7 exhibited significantly higher activity than the DMSO control at the viral inactivation stage, as shown in Figure 4B. AC#27 was also found to possess antiherpetic activity; however, this effect was seen at the post-entry stage (Figure 4F), indicating inhibition at later stages of the viral infection. The antiviral activity of AC#7 was found to be most effective when added to the virus prior to addition to the cells. No antiviral activity was observed when cells were preincubated with AC#7 (at 10 µg/mL) prior to HSV-1 infection (Figure S2). Based on the obtained data, the activity exhibited by AC#7 reflects the hypothesized mechanism of action predicted by the molecular docking study. Therefore, AC#7 was selected for further testing using a dose–response assay.

#### 2.2.3. Estimating the Efficacy of ACs by Plaque Reduction Assay

**Figure 5:**
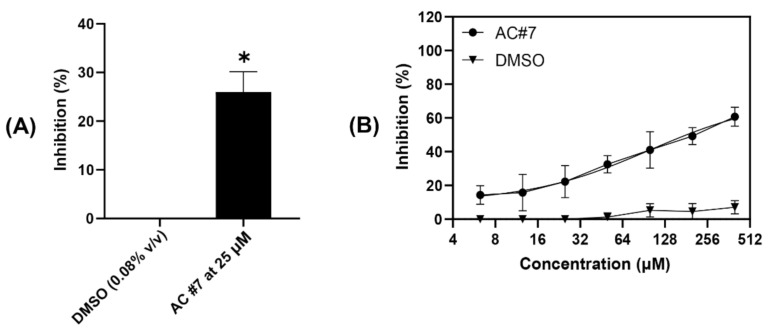
Quantification of the antiviral activity of AC #7. The antiviral activity of AC#7 was investigated by plaque reduction assay in African green monkey kidney cell line (VERO). (**A**) AC#7 demonstrated significantly high antiviral activity at a concentration of 25 µM compared to the dimethyl sulfoxide (DMSO) control (0.08% *v*/*v*). (**B**) AC#7 exhibited antiviral activity in a dose-dependent manner compared with the DMSO controls. The DMSO controls were tested at concentrations of 1.33%, 0.67%, 0.33%, 0.16%, 0.08%, 0.04%, and 0.02% (*v*/*v*), which are the concentrations at which AC#7 was dissolved in Dulbecco’s Modified Eagle Medium (DMEM) media. The plaque reduction assay was performed three times with duplicates. Means of the three experiments are plotted with standard error of the mean. One-sample t-test was applied to compare the antiviral activity of the DMSO control and AC#7 (* *p* < 0.05).

To visualize and evaluate the activity of AC#7, a plaque reduction assay was performed at concentrations from 400 µM (133.33 µg/mL) to 6.25 µM (2 µg/mL) using two-fold serial dilutions. Cells treated with DMSO only at concentrations ranging from 1.33% (*v*/*v*) to 0.02% (*v*/*v*) were used as a solvent control, and the results are plotted in Figure 5. AC#7 significantly reduced the number of virus-induced plaques compared to the DMSO control at a concentration of 25 µM (Figure 5A). This is consistent with the results obtained from the CPE assay, in which the AC was tested at a concentration of 30 µM (10 µg/mL) (Figure 4B). Notably, AC#7 demonstrated a dose-dependent antiviral response when pre-incubated with the virus compared to the DMSO control (Figure 5B).

**Figure 6:**
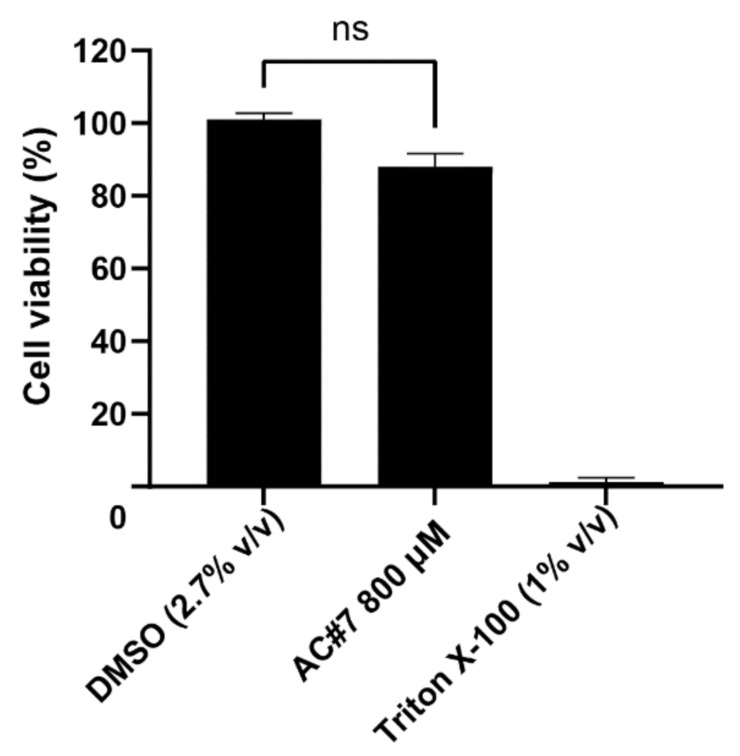
Toxicity of AC#7 in VERO cells at 800 µM. The cytotoxicity of AC#7 was tested by incubating Vero cells with AC#7 (800 µM) for 2 h. AC#7 was then removed, and cells were incubated in fresh media for a further 46 h before cell viability was evaluated using water-soluble tetrazolium 1. Cells without any treatment were included as cell-only control and used to calculate cell viability. Cells treated with dimethyl sulfoxide (DMSO) (2.7% *v*/*v*), corresponding to the solvent concentration in the tested samples, were used as the solvent control. The wells treated with 1% (*v*/*v*) Triton X-100 were used as a negative control. The cytotoxicity assay was performed three times with duplicates. Means of the three experiments are plotted with standard error of the mean. Wilcoxon test was applied to compare the cytotoxicity of DMSO control and AC#7 (ns indicates not significant, *p* > 0.05).

The EC_50_ value of AC#7 was 183 µM (61 µg/mL), as calculated based on the asymmetric nonlinear regression model (R^2^ = 0.84). To determine the therapeutic index (TI), the cytotoxicity of AC#7 was further tested at a concentration of 800 µM. Cell viability was measured 46 h after the 2 h of exposure to AC#7. Negligible toxicity to VERO cells was found, as illustrated in Figure 6. Thus, the TI of AC#7 is estimated to be >4.37, which suggests this molecule could serve as a potential lead compound for future antiviral drug development [43,44].

For comparison, the standard antiherpetic drug ACV was tested in parallel to AC#7. ACV was added post-HSV infection due to its inhibitory mechanism at the viral DNA replication stage (Figure S3). The EC_50_ of ACV was 0.77 µM, determined using the same asymmetric nonlinear regression model (R^2^ > 0.99). This is consistent with previously published in vitro results obtained with VERO cells in which the calculated EC_50_ for ACV lies between 0.3 and 4.3 µM [45,46].

## 3. Discussion

The basis of this study lies in the well-understood interactions between HSV-1 envelope glycoprotein D and its cellular receptors [18,19]. The wild-type strains of HSV-1 exploit either nectin-1 or HVEM as entry receptors to infect host cells, depending on the cell type [47,48]. For key target cells, neurons and human keratinocytes, nectin-1 has been reported as the primary receptor and is therefore the most important. Alternatively, HVEM functions as the main receptor in nectin-1-deficient cell lines, with similar infectivity [47,48,49]. Thus, it may be important for antiviral compounds to inhibit both nectin-1 and HVEM binding sites on gD to be considered potential therapeutic agents. The interaction between HVEM and gD forms an N-terminal hairpin structure that masks the nectin-1 binding site, whereas the binding between nectin-1 and gD blocks the accessibility of the HVEM [16]. Therefore, molecular docking must be performed on multiple regions of the glycoprotein, a requirement that entails significant computational resources to screen the large compound library of over 500,000 molecules used in this study. To overcome this obstacle, an innovative docking strategy was applied, resulting in the selection of eight compounds with predicted affinity to both binding sites in silico.

Among the eight chosen molecules, AC#7, [1-(1-benzofuran-2-yl)-2-[(5Z)-2H,6H,7H,8H-[1,3] dioxolo[4,5-g] isoquinolinelin-5-ylidene]ethenone] was identified as a weak HSV-1 inhibitor. The Gibbs free energy values generated by the docking software for AC#7 on both sites were −8.2 kcal/M and −8.6 kcal/M for the HVEM and nectin-1 sites, respectively. However, affinity with gD is not necessarily correlated with the potency of antiviral activity. A number of aptamers that have been reported to demonstrate strong affinity with gD at nanomolar concentrations using surface plasmon resonance were found to possess insignificant antiviral activity in vitro [50]. Therefore, some highly ranked hits, such as AC#28 and #29, may still bind gD, although they failed to show antiherpetic activity in the in vitro assays.

Two weak antiviral ligands were identified in this study; however, only AC#7 demonstrated activity at the early stage of infection, aligning with the in silico results. AC#7 is predicted to interact with nine residues in the HVEM binding interface via hydrophobic contacts and nine residues in the nectin-1 binding interface via hydrophobic contacts and two hydrogen bonds. Notably, the interaction at the nectin-1 interface involves amino acid residue Y38, which has been identified as critical for viral entry via the nectin-1 receptor [51,52,53]. Additionally, there are no overlapping residues between the binding interfaces, suggesting that the compound can bind to both sites simultaneously. However, although AC#7 exhibited low antiviral activity in the early stage of infection, further tests are required to confirm the interaction between this compound and glycoprotein D. Techniques such as surface plasmon resonance could provide deeper insights into the mechanism of action at a molecular level and could guide chemical modifications to improve the antiviral efficacy of this compound.

The compound 9-((2-Hydroxyethoxy) methyl) guanine, commonly known as ACV, is the gold standard in the treatment of HSV-1 infection, with an EC_50_ of around 1 µM in vitro [54]. ACV is a typical nucleoside analogue that acts after viral entry, requiring phosphorylation by virally encoded thymidine kinase to prevent viral DNA elongation, resulting in a shorter duration of infection and relief from symptoms [10,55]. Other antiherpetic drugs, including nucleosides, nucleotides, and pyrophosphate analogues, all target viral replication and hence fail to eliminate the lifelong latent infection caused by HSV-1 [10]. Docosanol is the only approved herpesvirus entry inhibitor and directly interferes with the host-cell surface phospholipids [56]. However, drugs targeting host cells may lead to higher toxicity and lower selectivity compared to compounds that interact specifically with viral components [10,57]. Compared to these drugs, AC#7 demonstrated a distinct mechanism, reducing HSV-1 infection at an early stage. This unique activity of AC#7 can be used to exploit this molecule as the basic structure for future drug modification.

Although AC#7 acts at the early stage of infection with an acceptable selectivity (therapeutical index >4) [44], its low antiviral activity remains the main drawback for its consideration as a potential antiviral agent. This may be explained by several reasons. Firstly, it has proven challenging for small molecular compounds to inhibit high-affinity interactions between proteins with contributions from either continuous or discontinuous sites in their respective protein structures [58,59,60]. Another explanation is the potential interaction between these ligands and other viral or cellular components. Given that molecular docking does not account for the complex environment of in vitro conditions, unknown off-target effects are a limitation of virtual screening [61].

Discovery of structures with low activity can lead to the identification of potent compounds via medicinal chemistry approaches. As an example, the HSV-1 ribonucleotide reductase inhibitor BILD 1633 SE demonstrated higher antiviral activity than ACV in vitro and exhibited a potent therapeutic effect against ACV-resistant HSV-1 infections in vivo [62]. This antiherpetic agent was designed via extensive drug modification and optimization of a series of peptides with initial EC_50_ values between 30 and 780 µM in vitro [63,64,65]. The drug improvement process of BILD1633 SE demonstrates the feasibility of improving the potency of molecules via medicinal chemistry approaches [62,63,64,65]. More recently, a chemical-modification design strategy targeting the cysteine-3-like protease (3CL^pro^) enzyme of severe acute respiratory syndrome coronavirus (SARS-CoV), has given rise to the novel antiviral PF-00835231, the main component of Paxlovid [66]. A previously identified inhibitor of human rhinovirus (rupintrivir) was used as the basis of the study; however, the drug and other modified versions produced weak to undetectable inhibition of SARS-CoV 3CL^pro^. Consequently, structural binding data from co-crystallography experiments were used to guide chemical modifications, resulting in the production of PF-00835231. Although initially intended for SARS-CoV, this compound demonstrates promising activity against SARS-CoV-2 and is currently being studied in clinical trials [67]. Based on these examples, a similar pathway could be applied to AC#7 to engineer superior antiherpetic compounds.

Natural compounds are an important resource for modern drug development. More than 100 small molecules with diverse antiherpetic activities have been identified from a wide range of organisms [8,43,68,69]. AC#7 was originally derived from glycyrrhiza glabra, which has been found to have versatile bioactivity, including antiviral activity [70,71,72,73]. This molecule contains benzofuran and isoquinoline elements (Figure 2), which have both been found to possess antiviral abilities [74,75,76]. To the best of our knowledge, this is the first report of antiherpetic activity of a molecule with both benzofuran and isoquinoline elements in vitro, suggesting a promising foundation for designing a new generation of antiviral drugs via medicinal chemistry.

The fact that AC#7 reduced HSV-1 at the early stage of infection in a dose-dependent manner shows that this compound likely acts on the viral surface glycoprotein, gD, as predicted by the molecular docking analysis. Identification of a new natural molecule with an antiviral mode of action different from that of ACV provides a fresh basis for subsequent drug development and optimization.

## 4. Materials and Methods

### 4.1. In Silico Screening

#### 4.1.1. Preparation of Natural Compound Library

A total of 527,209 compounds from five natural compound libraries (SuperNatural II [77], Phenol Explorer [78], Human Metabolome Database [79], Marine Natural Products [80], and ZINC Natural Products [81]) were downloaded in SDF format from the Miguel Hernandez University Molecular Docking site [82]. To prepare ligands for docking, each compound was edited with PyMOL to include polar hydrogens [83]. For the initial round of virtual screening, 3D coordinates were generated by converting files to MOL2 format with Marvin Suite 6.0 from ChemAxon [84]. Ligands were then energy-minimized using the universal force field (UFF) and converted to PDBQT format with Open Babel software v 3.1.1[85]. For the second round of screening, ligands were energy-minimized and converted from SDF to PDBQT format using the PyRx virtual screening tool [86].

#### 4.1.2. Preparation of Receptor Proteins

Glycoprotein D, the key surface protein of HSV-1 involved in viral attachment to host cells, was selected for molecular docking. The X-ray-derived crystal structures for gD complexed with HVEM (Protein Data Bank (PDB) ID: 1JMA) and in unliganded form (PDB ID: 2C36) were downloaded from the RCSB protein databank [18]. To prepare receptors for screening, files were edited using PyMOL to add polar hydrogens and to remove water molecules, ions, and ligands [83]. PDB files were converted to PDBQT format using the PyRx virtual screening tool [86].

#### 4.1.3. Virtual Screening on the HVEM Binding Site of gD

Virtual screening was performed using AutoDock Vina molecular docking software [41]. A targeted docking approach was employed by defining a search space containing residues involved in the interaction with the native HVEM receptor [18]. The crystal structure of gD bound to HVEM (1JMA) was used as the docking model, and the search space was defined as follows: center: coordinates (*x,y,z*): −27.284, 50.152, −6.099; dimensions (*x,y,z*): 15.364, 23.087, 22.147. A flexible docking procedure was adopted to account for changes in the receptor conformation. The gD residues defined as flexible were: C15, R24, V25, E27, A28, and C29.

To identify optimal docking conformations whilst minimizing the number of computational resources required, the protocol was conducted in two stages. The initial round of screening was performed with all 527,209 ligands. For each ligand, the number of docked conformations (modes) was set to 10, and the number of runs (exhaustiveness) was set to 20. A secondary, more thorough screen was then conducted with the top 10,000 ligands, with the number of runs increased to 80. As some ligands produced identical docking scores, the number of compounds screened in this round was 10,897.

#### 4.1.4. ADMET Analysis

To filter for compounds with favorable absorption, distribution, metabolism, excretion, and toxicity (ADMET) properties, ligands ranked within the top 25 scores from the secondary screen were assessed (*n* = 3893). The OSIRIS property explorer was used to compare the properties of each ligand to currently traded drugs and predict their toxicity based on data from the Registry of Toxic Effects of Chemical Substances (RTECS) database [42]. Ligands were filtered for the absence of toxic fragments (mutagenic, tumorigenic, irritant, or reproductive effects), molecular weight (MW) < 500 Da, hydrophilicity (cLogP) < 5, solubility (logS) >−6, topological surface area (TPSA) < 120Å^2^, drug likeness > 0, and overall drug score >0.5.

#### 4.1.5. Redocking Selected Compounds on the Nectin-1 Binding Site of gD

As nectin-1 is a primary receptor of gD and occupies a binding site distinct from that of HVEM, the ligands ranked with the top 25 scores, filtered for favorable ADMET profiles and commercial availability, were redocked against the nectin-1 binding site of gD (*n* = 26). For this docking experiment, an unliganded X-ray-derived crystal structure of gD was used due to its high resolution of 2.11Å (PDB ID: 2C36) [19]. The search space was selected based on a region of interacting residues at the nectin-1 binding interface known as surface patch 2 [87]. The following residues were defined as flexible: V37, Y38, H39, Q132, V214, D215, I217, M219, L220, R222, and F223. The search space was defined as follows: center coordinates (*x,y,z*): 60.637, 42.391, 98.991; dimensions (*x,y,z*): 20.428, 24.261, 15.139. As with the virtual screen against the HVEM binding site, the number of docked conformations was set to 10, and the number of runs was set to 80.

The docking results were visualized using PyMOL. Residues involved in hydrogen-bonding and hydrophobic interactions were plotted using LigPlot^+^ software [88].

### 4.2. In Vitro Validation

#### 4.2.1. Chemicals

A total of 8 synthetic purified ACs with docking scores ≤−8.2 for the HVEM site and ≤−7.6 for the nectin-1 site and favorable ADMET properties were selected for in vitro screening. All ACs were purchased from VITAS-M laboratory, USA, as listed in Table 1. Their molecular structures are illustrated by ChemDraw 18.2 (PerkinElmer). Each compound was dissolved in molecular-grade dimethyl sulfoxide (DMSO) (Sigma, Melbourne, Australia) to a final concentration of 10 mg/mL and kept at −20 °C until use.

#### 4.2.2. Cells and Virus

HSV-1 strain F was used in all antiviral assays in this study. African green monkey epithelial kidney (VERO) cells were cultured in Dulbecco’s modified Eagle medium (DMEM) (Lonza, Thermo Fisher Scientific, Walkersville, MD, USA) supplemented with 10% (*v*/*v*) fetal bovine serum (FBS) and 1% (*v*/*v*) penicillin streptomycin (Gibco, Thermo Fisher Scientific, Scoresby, VIC, Australia) at 37 °C under 5% CO_2_. HSV-1 was propagated in VERO cells and titrated by plaque assay as described in [8]. Viral stocks were aliquoted and kept at −80 °C until use.

#### 4.2.3. Cytotoxicity of ACs

The cytotoxicity of each AC was determined using WST-1 reagent (Sigma, Australia) as described previously [43]. VERO cells were seeded in 96-well plates at a density of 10,000 cells per well for 24 h at 37 °C under 5% CO_2_. Then, the ACs were 10-fold serially diluted in DMEM supplemented with 2% (*v*/*v*) FBS and 1% (*v*/*v*) penicillin streptomycin to concentrations of 100 µg/mL, 10 µg/mL, 1 µg/mL, and 0.1 µg/mL and incubated with the cells for 24 h at 37 °C. After incubation, cells were washed with DMEM with 2% FBS and 1% (*v*/*v*) penicillin streptomycin once and incubated with 10% (*v*/*v*) WST-1 for 2 h at 37 °C under 5% CO_2_. To determine cell viability, plates were read by a SpectraMAX iD3 plate reader (Molecular Devices) at 450 nm. The absorbance at 620 nm was used as a reference. Cells treated with DMSO only were included as solvent controls, and untreated cells were included as cell-only control. Wells containing DMEM without cells were prepared as blanks. The cytotoxicity of each AC was calculated by comparing treated cells with the DMSO control Equation (1). The experiment was performed in quadruplicate.

(1) $$1=\frac{{\mathrm{AC}}_{A450}-{\mathrm{Blank}}_{A450}}{{\mathrm{Cell}\;\mathrm{control}}_{A450}-{\mathrm{Blank}}_{A450}}\times 100\%=\mathrm{Cell}\;\mathrm{viability}\;\%$$

#### 4.2.4. Time of Addition Assay

A cytopathic effect (CPE) inhibition assay was applied to screen for the antiviral activity of selected ACs at different time points. VERO cells were seeded in 96-well plates at a density of 10,000 cells per well for 24 h before the addition of virus at 37 °C under 5% CO_2_. The initial screening was conducted at a multiplicity of infection (MOI) = 0.01; then, the active compounds were tested with a higher MO1 = 5 to confirm their activity. ACs were tested at 10 µg/mL or 1 µg/mL depending on their cytotoxicity.

To identify compounds with antiviral activity, virus and ACs were added at different time points: viral inactivation (preincubation of the virus with ACs for 1 h prior to addition to cells at 37 °C under 5% CO_2_), attachment and entry (ACs and virus were added to cells simultaneously), and post-entry stages (ACs were added 1 h after HSV-1 infection of cells and were kept during the entire 48 h incubation at 37 °C under 5% CO_2_). After infection, the cells were incubated for another 48 h in DMEM with 2% FBS before addition of WST-1 to quantify cell viability. Three controls were included: Solvent controls were prepared by diluting DMSO at concentration of 0.1% (*v*/*v*) in DMEM without ACs and added to the cells at different time points corresponding to the samples. Cells cultured with DMEM only were prepared as the cell control, and cells infected with virus without DMSO were included as the virus control. The initial antiviral activity of each AC was calculated using Equation (2) as described in [89], with modifications. The initial screening (with MOI = 0.01) was performed once in quadruplicate, and the secondary experiments (with MOI = 5) were conducted four times in triplicate. The ACs that demonstrated CPE inhibition were further analyzed by plaque reduction assay.

(2) $$2=\frac{{\mathrm{AC}}_{A450}-{\mathrm{Viral}\;\mathrm{control}}_{A450}}{{\mathrm{Cell}\;\mathrm{control}}_{A450}-{\mathrm{Viral}\;\mathrm{control}}_{A450}}\times 100\%=\mathrm{Inhibition}\;\%$$

#### 4.2.5. Plaque Reduction Dose–Response Assay

The antiviral activity of selected ACs on VERO cells was quantitatively determined by plaque reduction assay as described in [43], with modifications. VERO cells were seeded in 24-well plates (50,000 cells/well) for 24 h. The virus was diluted to 50 plaque-forming units (PFU) per 0.2 mL and preincubated with the ACs at different concentrations (400, 200, 50, 25, 12.5, and 6.25 µM) or DMSO controls (1.33%, 0.67%, 0.33%, 0.16%, 0.08%, 0.04%, and 0.02%, *v*/*v*) for 1 h before being added to the cell monolayer. Then, the cells were incubated with the mixture for 1 h at 37 °C under 5% CO_2_. After removing the inoculum, the cells were rinsed with PBS and overlayed with DMEM containing 1.6% carboxymethyl cellulose (CMC) and 1% FBS. After incubation for 48 h, the cells were fixed with methanol and stained with 0.5% crystal violet (Sigma, Australia). The number of plaques was counted manually using upright microscopy (Olympus, Tokyo Japan). Cells infected with virus without DMSO were included as the virus control. The antiviral activity was calculate using Equation (3) [90], and the EC_50_ values were determined by linear regression analysis of the dose–response curve.

(3) $$\;3=\frac{{\mathrm{Plaque}\;\mathrm{number}}_{\mathrm{Viral}}-{\mathrm{Plaque}\;\mathrm{number}}_{\mathrm{AC}}}{{\mathrm{Plaque}\;\mathrm{number}}_{\mathrm{Viral}}}\times 100\%=\mathrm{Inhibition}\;\%$$

#### 4.2.6. Toxicity of AC#7 on VERO Cells at High Concentrations

Since AC#7 did not exhibit cytotoxicity to VERO cells in the plaque reduction assay at a concentration of 400 µM, its toxicity was further tested at 800 µM in 96 well-plates. Due to stock limitation, 800 µM was the highest concentration that could be tested. Briefly, VERO cells were seeded in 96-well plates at a density of 10,000 cells per well for 24 h at 37 °C under 5% CO_2_. Then, the cells were exposed to AC#7 at a concentration of 800 µM or controls for 2 h at 37 °C under 5% CO_2_. AC#7 or controls were then removed and replaced with fresh DMEM with 2% FBS and 1% penicillin streptomycin. Cells were incubated for 46 h at 37 °C under 5% CO_2_. After incubation, cells were washed with DMEM once and incubated with 10% (*v*/*v*) WST-1 for 2 h at 37 °C under 5% CO_2_. Cells treated with DMSO at 2.7% (*v*/*v*), corresponding to the solvent used to dissolve 800 µM AC#7 in DMEM, were included as solvent control. Cells treated with Triton X-100 at 1% (*v*/*v*) (Sigma, Australia) were included as negative control, and untreated cells were included as cell-only controls. Then, the cell viability of AC#7 was calculated using Equation (1). Wells containing DMEM without cells were prepared as blanks. The experiment was performed three times with duplicates.

### 4.3. Statistical Analysis

All statistical analyses were performed using GraphPad Prism 9 software (GraphPad Software, Inc., San Diego, CA, USA). Linear regression curves were obtained using the five-parameter logistic sigmoidal model offered by the same software. Significance testing between multiple samples and controls was performed using Friedman test followed by a post-hoc Dunn’s multiple comparisons test. Wilcoxon matched test or *t*-tests were applied to compare the mean between two samples.

## 5. Conclusions

This study represents the first application of a large-scale structure-based virtual screen targeting the binding interfaces of HSV-1 glycoprotein D to identify inhibitors of early viral infection. Two out of eight selected compounds exhibited significant activity against HSV-1 infection in vitro. Notably, compound AC#7 demonstrated a mechanism of action consistent with inhibiting early-stage infection. Although the potency of AC#7 is lower than that of current first-line drugs, these data suggests that it could serve as a structural basis for further drug development. More importantly, this work supports the use of molecular docking as a powerful tool for identifying novel antiviral compounds. Future studies will expand on these computational methods with an aim to screen additional compound libraries, as well as other viral protein targets.

## Figures and Tables

**Table 1:** List of compounds selected for in vitro validation based on in silico predictions on the (herpes virus entry mediator) HVEM and nectin-1 binding interfaces of glycoprotein D.

ID	Database ID	Name	Empirical Formular	Molecular Weight	Docking Score on		Drug Score
					HVEM Site	Nectin-1 Site	
7	Sn00074072	1-(1-benzofuran-2-yl)-2-[(5Z)-2H,6H,7H,8H-[1,3]dioxolo [4,5-g]isoquinoline-5-ylidene]ethenone	C_20_H_15_NO_4_	333.34	−8.2	−8.6	0.58
10	Sn00115356	13-[3-(4-methylpiperazin-1-yl)-3-oxopropyl]-8,13-dihydroindolo[2′,3′:3,4] pyrido [2,1-b]quinazolin-5(7H)-one	C_26_H_27_N_5_O_2_	441.53	−8.2	−8.5	0.69
12	Sn00099520	(2S,5Ar,6Ar,9S,9Ar)-2,5a-dimethyl-9-((4-(isoquino-2-yl)piperazin-1-yl)methyl)octahydro-2H-oxireno[2′,3′:4,4a]naphtho[2,3-b]furan-8(9Bh)-one	C_24_H_33_N_3_O_3_	411.54	−8.5	−7.6	0.77
16	Sn00104387	(1Ar,2S,5Ar,6Ar,9S,9Ar,9Bs)-2,5a-dimethyl-9-((4-phenylpiperazin-1-yl)methyl)octahydro-2H-oxireno[2′,3′:4,4a]naphtho[2,3-b]furan-8(9Bh)-one	C_25_H_34_N_2_O_3_	410.56	−8.3	−7.6	0.74
17	Sn00104404	(1Ar,2S,5Ar,6Ar,9S,9Ar,9Bs)-9-( (4-( 5-chloro-2-methylphenyl)piperazin-1-yl)methyl)-2,5a-dimethyloctahydro-2H-oxireno[2′,3′:4,4a]naphtho[2,3-b]furan-8(9Bh)-one	C_26_H_35_ClN_2_O_3_	459.03	−8.4	−7.9	0.58
27	Zinc96221711	5-(7-Hydroxy-1H-benzofuro[3,2-b]pyrazolo[4,3-e]isoquino-4-yl)-1H-pyrrolo[3,2,1-ij]isoquinol-4(2H)-one	C_23_H_14_N_4_O_3_	394.39	−9.3	−9.4	0.53
28	Zinc96115494	N-((S)-5,11-dioxo-2,3,5,10,11,11a-hexahydro-1H-benzo[e]pyrrolo[1,2-a][1,4]diazepin-7-yl)-2-(3-oxoisoindolin-1-yl)acetamide	C_22_H_20_N_4_O_4_	404.43	−9.5	−8.6	0.76
29	Sn00346605	Arcyriaflavin A	C_20_H_11_N_3_O_2_	325.3	−9.1	−9.1	0.89

**Table 2:** Predicted interactions between (herpes virus entry mediator) HVEM and nectin-1 binding interfaces of glycoprotein D and compounds selected for in vitro validation.

ID	HVEM Binding Interface			Nectin-1 Binding Interface		
	No. of Interacting Residues	No. of H-bonds	Interacting Residues	No. of Interacting Residues	No. of H-bonds	Interacting Residues
7	9	0	M11, A12, P14, F17, L22, P23, V24, L25, Y234	9	2	Y38, H39, R134, D215, L220, P221, I296, P297, A303
10	11	1	M11, A12, P14, F17, L22, P23, V24, L25, D26, Q27, Y234	12	2	Y38, H39, R134, D215, M219, L220, P221, I296, P297, S298, I299, A303
12	9	0	M11, A12, P14, F17, P23, V24, L25, Q27, Y234	9	0	Y38, H39, R134, T213, D215, I299, D301, A302, A303
16	9	0	M11, A12, P14, F17, P23, V24, L25, Q27, Y234	9	0	Y38, H39, R134, T213, D215, I299, D301, A302, A303
17	11	0	M11, A12, D13, P14, F17, P23, V24, L25, D26, Q27, Y234	10	1	Y38, R134, D215, L220, P221, I296, P297, S298, I299, A303
27	9	2	A12, P14, N15, F17, R18, G19, L22, V24, L25	8	2	Y38, R134, D215, L220, P221, R222, I296, P297
28	13	3	M11, A12, D13, P14, F17, R18, G19, L22, P23, V24, L25, Q27, Y234	8	3	Y38, H39, R134, T213, D215, P221, A303, T304
29	9	1	M11, A12, P14, F17, L22, V24, L25, Q27, Y234	6	2	Y38, R134, D215, G218, L220, P221

**Table 3:** Cell viability of tested antiherpes active compounds with African green monkey kidney cell line (VERO).

ID	Highest Concentration with Cell Viability above 75%	Relative Cell Viability *	Test Concentration
7	>100 µg/mL	123.7% ± 4.8%	10 µg/mL
10	1 µg/mL	73.2% ± 5.9%	1 µg/mL
12	1 µg/mL	77.4% ± 4.2%	1 µg/mL
16	1 µg/mL	102.1% ± 16.7%	1 µg/mL
17	1 µg/mL	96.8% ± 7.1%	1 µg/mL
27	>100 µg/mL	105.9% ± 0.2%	10 µg/mL
28	>100 µg/mL	105.0% ± 0.5%	10 µg/mL
29	1 µg/mL	86.2% ± 14.8%	1 µg/mL

* Relative cell viability of ACs is presented as mean ± standard deviation.

## Data Availability

Data is contained within the article and Supplementary Materials.

## Supplementary Materials

The following are available online at https://www.mdpi.com/article/10.3390/ph15030361/s1, Figure S1: Preliminary screening of antiviral activity of eight select ACs; Figure S2: Investigating the antiviral activity of AC#7 when added to cells before HSV-1 infection; Figure S3: Inhibition of antiviral activity by ACV in the post-entry stage.
