# Supplementary material for: Identifying HSV-1 Inhibitors from Natural Compounds via Virtual Screening Targeting Surface Glycoprotein D
Source: Pharmaceuticals (Basel). 2022 Mar 16;15(3):361. doi: 10.3390/ph15030361 (PMC8955139; doi:10.3390/ph15030361)
Supplement: Supplementary file 1 [file pharmaceuticals-15-00361-s001.zip › pharmaceuticals-1595369-supplementary.pdf]

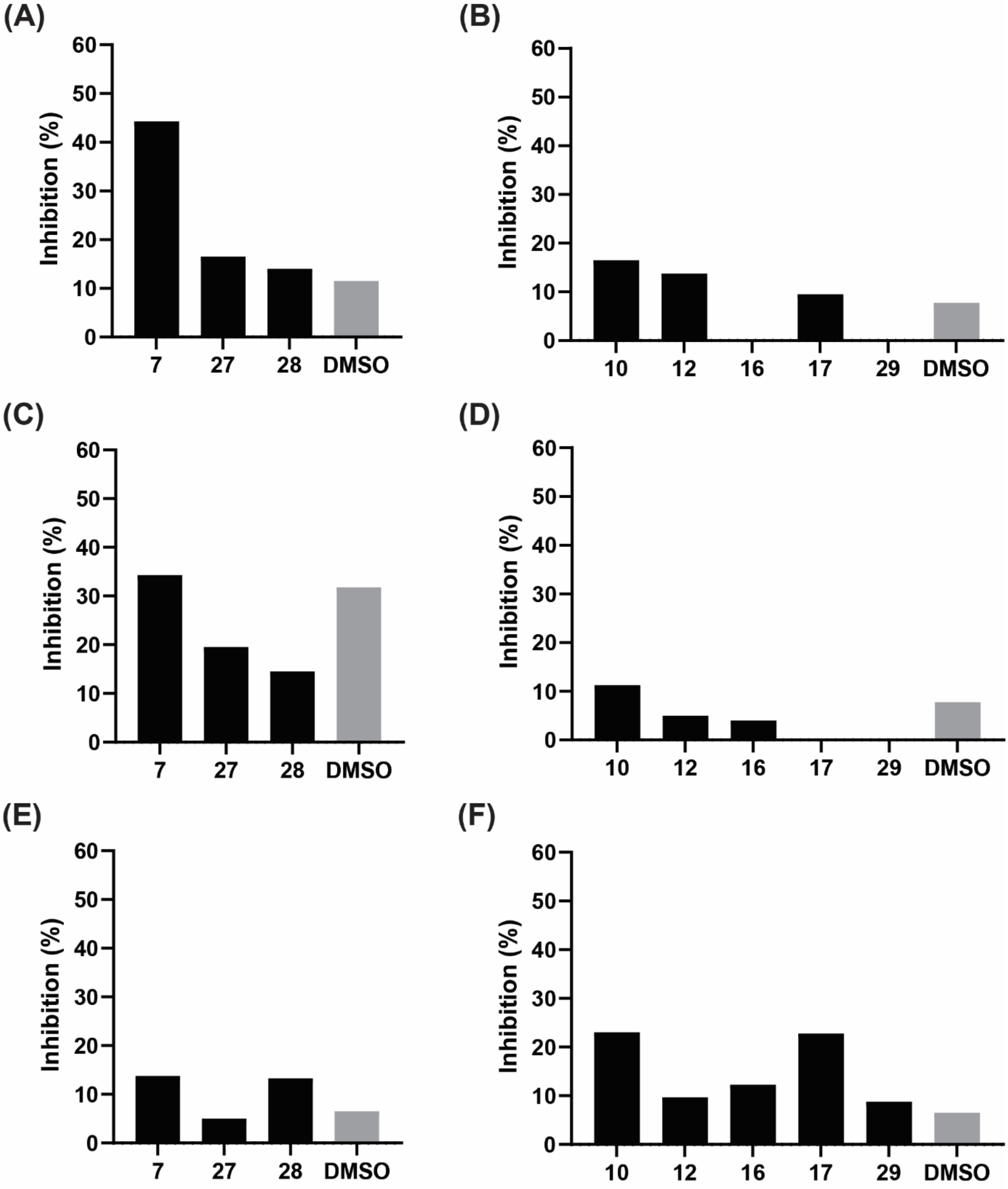

Figure S1. Preliminary screening for antiviral activity of eight select active compounds

The antiviral activity of active compounds (ACs) #7, #27 and #28 was tested at 10 µg/ml at the viral inactivation stage (A), viral attachment and entry stage (C), and post-entry stage (E). ACs #10, #12, #16, #17 and #29 were tested at 1 µg/ml in viral inactivation (B), attachment and entry (D) and post-entry (F) stages. The experiment was performed with Multiplicity of infection (MOI)=0.01 in four technical quadruplicates. Cells infected with the virus and treated with dimethyl sulfoxide (DMSO) corresponding to the concentration used for tested ACs were included as a solvent control. Plotted are the means of the technical quadruplicates.

(A)

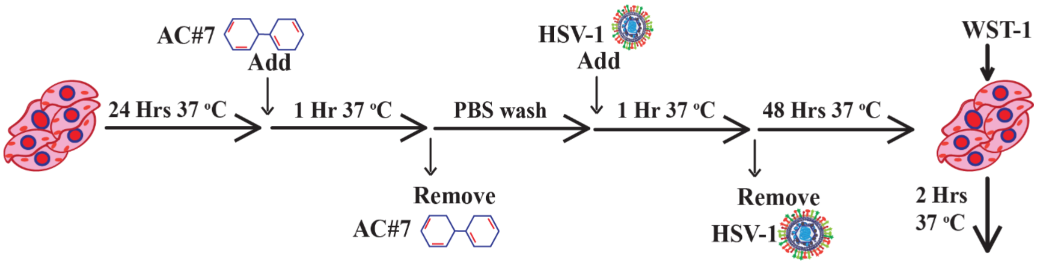

(B)

Description	Virus	DMSO	AC#7	Cell survival rate (%)	Inhition (%)
Cell control	-	-	-	100%	-
Viral control	MOI=5	-	-	7.7%±0.9%	-
Solvent control	MOI=5	0.1% (v/v)	-	7.0%±0.3%	0%
AC#7	MOI=5	0.1% (v/v)	10 µg/ml	7.7%±1.2%	0%

Figure S2. Investigating the antiviral activity of AC#7 when added to cells before Herpes Simplex Virus type 1 infection.

The schamatic diagram of the assay is illustrated in (A). AC#7 was added to a monolayer of African Green monky kidney (VERO) cells one hour before infection at a final concentration of 10 µg/ml, containing 0.1% (v/v) dimethyl sulfoxide (DMSO). After one hour of incubation at 37 °C, 5% CO2, AC#7 was removed, and the cells were washed with phosphate-buffered saline (PBS). Then, the cells were infected with HSV-1 at the multiplicity of infection (MOI)=5. After removing the innoculumn 1 hour post infection, the cells were incubated for another 48 hours at 37 °C, 5% CO2. Three controls listed in (B) were tested simultaneously.The experiment was performed in quadruplicates. The cells treated with AC#7 did not exhibit antiviral activity.

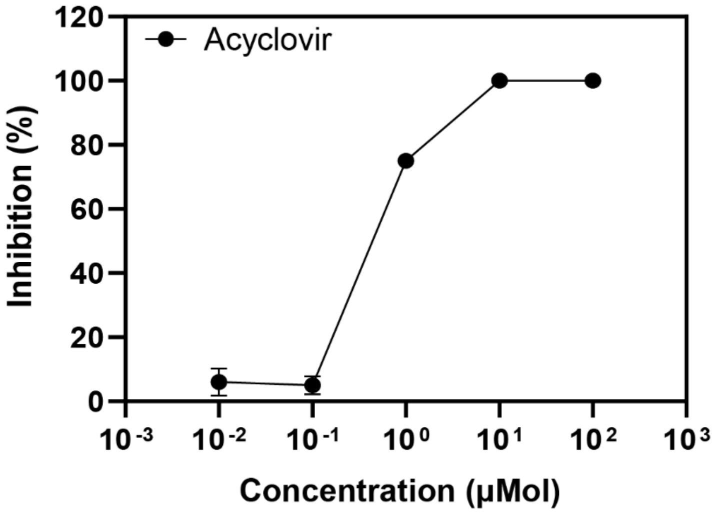

Figure S3. Inhibition of antiviral activity by acyclovir in the post-entry stage

As a positive control, the antiviral activity of acyclovir was investigated by plaque reduction assay in African Green monky kidney cells at the post-entry stage. Acyclovir was tested at concentrations of 100 µMol, 10 µMol, 1 µMol, 0.1 µMol, and 0.01 µMol. The calculated half maximal effective concentration (EC_{50}) of acyclovir in the post-entry stage was 0.77 µMol, (R^2 >0.99).
